# Preparation of 1D Hierarchical Material Mesosilica/Pal Composite and Its Performance in the Adsorption of Methyl Orange

**DOI:** 10.3390/ma11010164

**Published:** 2018-01-20

**Authors:** Mei Wu, Haifeng Han, Lingli Ni, Daiyun Song, Shuang Li, Tao Hu, Jinlong Jiang, Jing Chen

**Affiliations:** 1 Faculty of Chemical Engineering, Huaiyin Institute of Technology, Key Laboratory for Palygorskite Science and Applied Technology of Jiangsu Province, Huai’an 223003, China; linglini@hyit.edu.cn (L.N.); ssddyy@shu.edu.cn (D.S.); lishuang1248361263@163.com (S.L.); hutao@hyit.edu.cn (T.H.); jingchen@hyit.edu.cn (J.C.); 2 Huai’an Research Center of Chemical and Advanced Materials, Dalian Institute of Chemical Physics, Chinese Academy of Sciences, No. 19 Meigao Road, Wisdom Valley, Huai’an 223005, China; 3 Jiangsu HanbonSci. & Tech Co., Ltd., No. 1-9 Ji’xian Road, Economic Development Zone, Huai’an 223003, China; hhflicp@163.com

**Keywords:** adsorption, mesosilica, methyl orange, palygorskite

## Abstract

This paper highlights the synthesis of a one-dimensional (1D) hierarchical material mesosilica/palygorskite (Pal) composite and evaluates its adsorption performance for anionic dye methyl orange (MO) in comparison with Pal and Mobile crystalline material-41 (MCM-41). The Mesosilica/Pal composite is consisted of mesosilica coated Pal nanorods and prepared through a dual template approach using cetyltrimethyl ammonium bromide (CTAB) and Pal as soft and hard templates, respectively. The composition and structure of the resultant material was characterized by a scanning electron microscope (SEM), transmissionelectron microscopy (TEM), N_2_ adsorption-desorption analysis, small-angle X-Ray powder diffraction (XRD), and zeta potential measurement. Adsorption experiments were carried out with different absorbents at different contact times and pH levels. Compared with Pal and MCM-41, the mesosilica/Pal composite exhibited the best efficiency for MO adsorption. Its adsorption ratio is as high as 70.4%. Its adsorption equilibrium time is as short as 30 min. Results testify that the MO retention is promoted for the micro-mesoporous hierarchical structure and positive surface charge electrostatic interactions of the mesosilica/Pal composite. The regenerability of the mesosilica/Pal composite absorbent was also assessed. 1D morphology makes it facile to separate from aqueous solutions. It can be effortlessly recovered and reused for up to nine cycles.

## 1. Introduction

The colored wastewater generated from the extensive use of dyes has produced toxicological problems and has become a major environmental concern [1]. Among the various techniques developed for effectively removing toxic dyes, adsorption is considered a simple and facile method that is adaptive to the personal and portable use for water supply [2]. Considerable efforts have been devoted to the preparation of mesoporous silica-based (mesosilica) adsorbents because of their high surface area, ordered pore structure, and tunable pore diameters [3,4,5]. In addition, mesosilica adsorbents have the advantage of high thermo-stability due to their silica skeleton. However, conventional mesosilica materials are difficult to separate from aqueous solutions, especially when the particle size is at the nanoscale, at which adsorbents possess considerably larger surface areas and higher adsorption capacities [6].

In recent years, one-dimensional (1D) nano-materials, such as nanofibers, nanorods, and nanotubes, have been extensively studied for environmental applications owing to their advanced properties such as large surface to volume ratio, high efficiency, and appreciable physical and chemical properties, etc. [7,8,9,10]. 1D nano-material absorbents possess a maximum surface area owing to their nanosize diameter with a high surface-to-volume ratio [11]. At the same time, their length ranges at several micrometers, at which they can be easily separated from solution by conventional methods such as filtration and centrifugation, etc. [12]. Therefore, aside from promoting dye retention with mesoporous channels, mesosilica adsorbents with 1D morphology can solve the recovery problem in liquid samples.

1D mesosilica materials can be synthesized through several methods, such as the sol-gel process [13], spray-drying method [14], laser deposition [15], and solvent evaporation techniques [16]. However, these techniques are typically hindered by their complex processes and high-cost templates. Palygorskite (Pal) is a hydrated magnesium aluminum silicate that exists in nature as a 1D fibrous mineral. The diameter of Pal is generally in the nanosize range of approximately 10 nm, and its length ranges from several hundred nanometers to several micrometers. In view of its 1D morphology and low cost, lots of 1D structures and mesoporous materials such as amorphous carbon nanotubes and mesoporous carbon nanosheets [17,18] have been prepared using Pal as a hard template. Additionally, in our previous work, Pal was used simultaneously as hard templates and a silica source to prepare amorphous carbon nanotubes and SBA-15 mesoporous materials [19]. Usually, the clay hard template should be removed with hydrofluoric acid (HF) treatment. Nevertheless, HF is a strong toxic and corrosive acid. Sacrificial clay templates cannot be recycled, and the waste leaching solution presents another environmental hazard. Actually, Pal itself is a good candidate to remove ionic dyes due to its rough surface, relatively high surface area, and moderate cation exchange capacity [20]. Even if compared with other clay minerals, Pal has superiorities in salt resistance and a rapid hydration rate. However, Pal cannot provide abundant active adsorption sites due to its aggregate crystal bundles, micropore channels, and low specific surface area.

In the present study, a 1D mesosilica/Pal composite which consisted of mesosilica coated Pal was prepared for the removal of methyl orange (MO) from solution. The Pal hard template was retained as a component, and a micro-mesoporous hierarchical structure and 1D nanorod morphology were generated. The advantages of Pal, consisting of a 1D morphology and superior adsorption performance, were extensively and innovatively taken simultaneously as the hard template and part of absorbent in this report. The micro-mesoporous hierarchical structure of 1D mesosilica/Pal composite will improve the retention time of MO and promote the MO adsorption ratio. Furthermore, its 1D nanorod morphology makes it facile to be recovered from the solution and improves its recyclability. In comparison with Pal original clay and MCM-41 mesoporous zeolite, the mesosilica/Pal composite absorbent shows the best adsorption efficiency. Results testified that it possesses superior regenerability and can be reused for up to nine cycles in the removal of MO.

## 2. Experimental Section

### 2.1. Preparation of Mesosilica/Pal Composite

**Scheme 1:**
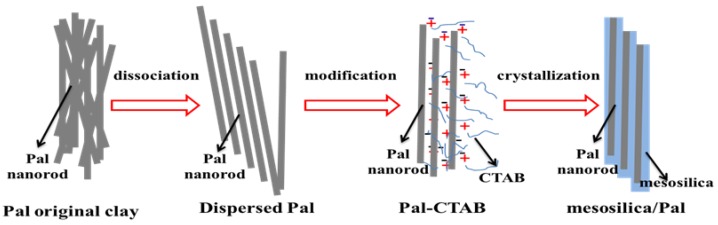
Schematic illustration of mesosilica/Pal composite preparation.

The preparation of the mesosilica/Pal composite is illustrated in Scheme 1. As shown, the fabrication process includes three steps. Firstly, the Pal original clay was purified and dissociated to the scattered Pal nanorod suspension. Secondly, the surfactant cetyltrimethyl ammonium bromide (CTAB) was used to modify the Pal nanorod, resulting in the dual-template Pal-CTAB. Thirdly, under the structure-direction effect of the Pal-CTAB, the mesosilica/Pal composite was synthesized through hydrothermal crystallization.

#### 2.1.1. Purifying and Dissociation of Pal Original Clay

To purify Pal, 5 g of the original clay was treated with 100 mL of hydrochloride acid (1 mol/L) for 48 h. The upper white Pal cake collected through high-speed centrifugation was neutralized by repeated washing, followed by freeze-drying for 12 h. In this way, loose Pal powder was obtained. Then, a given amount of Pal powder was roll extruded five times, followed by beat and ultrasonic dispersion in water. Finally, the scattered Pal nanorod suspension was obtained.

#### 2.1.2. Fabrication of Dual-Template Pal-CTAB

A total of 0.75 g of CTAB was dissolved in 10 mL deionized water and added to the scattered Pal nanorod suspension. The mixture was refluxed in an oil bath for 12 h at 80 °C. The obtained suspension was the dual-template Pal-CTAB without further treatment.

#### 2.1.3. Preparation of Mesosilica/Pal Composite

A total of 9 g of ethanol and 4.8 g of ammonia water were added to the Pal-CTAB template. After stirring for 15 min, 1.5 g of tetraethyl orthosilicate (TEOS) was dropwise added to the solution. Then, the as-made mesosilica/Pal composite was crystallized for 48 h at 100 °C, washed, dried, and finally calcined for 5 h at 550 °C. For comparison, MCM-41 was prepared under the same condition but without the use of Pal as the hard template.

### 2.2. Characterization

Small-angle X-ray diffraction (XRD) was applied to characterize the long-range order of all samples. The XRD patterns were recorded on a D8-Discover diffractometer (Bruker) with Cu Kα radiation (40 kV, 40 mA). ICP-OES (Inductively Coupled Plasma Optical Emission Spectrometry) analyses were performed on a Thermo Electron IRIS Intrepid II XSP instrument (thermo fisher scientific, UK). The N_2_ adsorption and desorption isotherms were measured with the Micromeritics TriStar II 3020 (Micromeritics, Norcross, GA, USA) at 77 K. The samples were out-gassed for 3 h at 300 °C under N_2_ atmosphere before the measurements were recorded. The total surface area was calculated through the Brunauer–Emmett–Teller (BET) method. The total pore volume was calculated from the desorption branch of the isotherm at P/Po = 0.99 under the assumption of complete pore saturation. The t-plot method was adopted to evaluate the mesopore volume. The scanning electron microscopy (SEM) images of the samples were collected with an S-3000N scanning electron microscope (Hitachi, Tokyo, Japan) operated at 20 kV. The transmission electron microscopy (TEM) micrographs were obtained by using a JEM 2010 (Japan electronic materials, Tokyo, Japan) with an accelerating voltage of 200 kV. The infrared (IR) spectra of all samples were obtained with a Nicolet 5700 spectrometer (Thermo electron scientific, USA) at a resolution of 4 cm^−1^. The zeta potential was measured with a Malvern Zetasizer Nano ZS analyzer (Malvern Instrument Ltd., UK) equipped with a multipurpose autotitrator (model MPT-2, Marvern Instruments, UK).

### 2.3. Evaluation of Adsorption Performance

The adsorption performance of the mesosilica/Pal composite was evaluated by conducting MO adsorption experiments in comparison with Pal and MCM-41. The contact time, pH, and recyclability of the mesosilica/Pal composite were investigated. An amount of 50 mg of the mesosilica/Pal composite was added to 20 mL MO solutions (200 mg·L^−1^) in 100 mL conical flasks. After capping, the conical flasks were placed at room temperature and gently stirred for a specific time period at the speed of 100 rpm. They were replicated three times in the different experiments when MO adsorption was assessed. In these experiments, all assessed factors were checked in duplicate. The effect of pH on dye removal was studied over a pH range of 3–9. The initial pH of the dye solution was adjusted by adding 1 mol/L solution of HCl or NaOH. The mesosilica/Pal composite was regenerated by calcination at 500 °C for 2 h followed by separation from the waste solution.

The MO concentration (C_MO_) and MO absorbance (A_MO_) in the solutions were determined through a UV-vis spectrophotometer with a Hewlett Packard 8453 spectrophotometer at the wavelength of 470 nm. The adsorption ratio (q_e_) was defined as the percentage removal of the adsorbate and calculated as Formula (1). The adsorption capacity (Q_ads_, mg/g) was defined as the mass of MO adsorbed per amount of absorbents and calculated as Formula (2).

q_e_ = (C_0_ − C_e_)/C_0_ × 100% (1)

Q_ads_ = (C_0_ − C_e_) × V_0_/m_e_ (2)

where C_0_ and C_e_ are the initial and equilibrium concentrations of MO (mg/L), respectively; V_0_ is the volume of solution; and m_e_ is the amount of absorbents.

## 3. Result and Discussion

### 3.1. Characterization of Mesosilica/Pal Composite

The micro-mesoporous hierarchical structure and 1D morphology of the prepared mesosilica/Pal composite were examined by conducting small-angle XRD measurements, SEM analysis, TEM observations, and nitrogen adsorption–desorption measurements.

**Figure 1:**
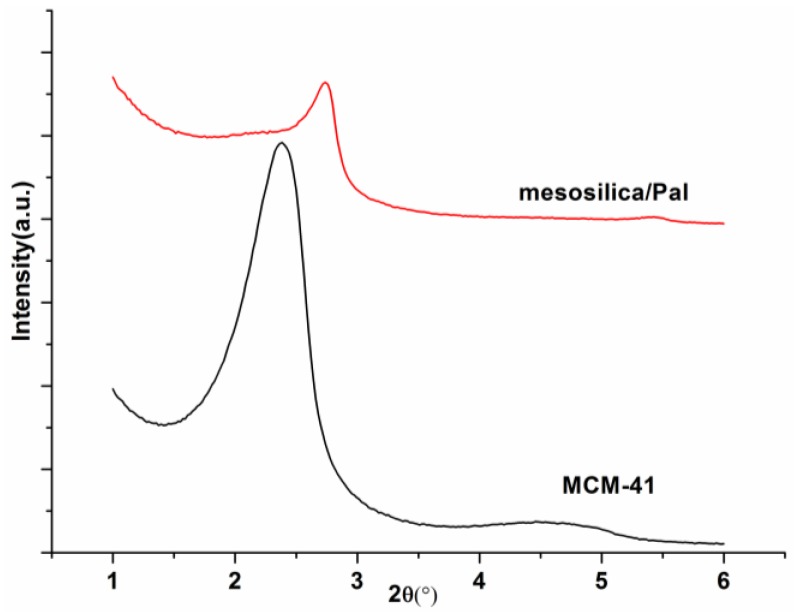
The small-angle XRD of the mesosilica/Pal composite and MCM-41.

As shown in Figure 1, MCM-41 exhibited well-resolved diffraction peaks at 2θ = 2.4° indexed to the (100) planes. Similarly, an obvious diffraction peak corresponding to the periodic meso-structure was observed at 2θ = 2.6° for the mesosilica/Pal composite. The appearance of these peaks implies the presence of hexagonal shaped pores in MCM-41 and the mesosilica/Pal composite [21]. However, the intensity of this relevant diffraction peak markedly decreased, as evidenced by the loss of the long-range order. This finding indicates that although periodic mesopores can be generated under the structure direction of CTAB, the Pal hard template would hamper the self-assembly of surfactant micelles and silica precursors. The slight shift of the diffraction peaks of the mesosilica/Pal composite toward the larger 2θ angles relating to MCM-41 indicated a slight shrinkage of the cell dimension [22]. The mesosilica/Pal composite only displays peaks at low angles and no any additional peaks were observed at higher angles. It demonstrates that the arrangement of atoms within the walls of the mesosilica/Pal composite is basically amorphous and the crystalline structure of Pal is collapsed totally [23].

**Figure 2:**
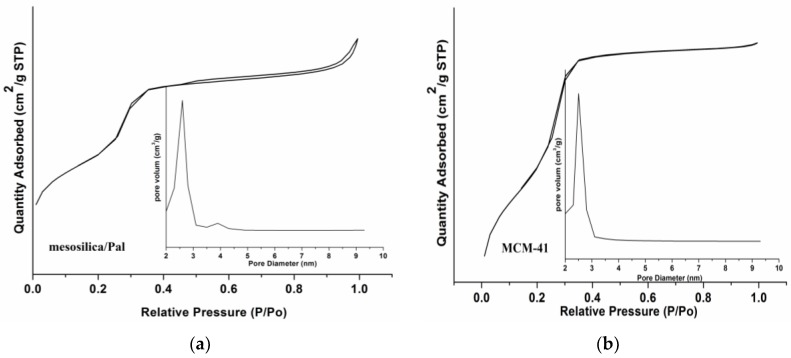
The N_2_ adsorption and desorption isotherm curves and pore diameter distributions of the mesosilica/Pal composite and MCM-41, (**a**) mesosilica/Pal; (**b**) MCM-41.

The nitrogen adsorption–desorption isotherms and pore diameter distributions of the mesosilica/Pal composite and MCM-41 are shown in Figure 2. Table 1 provides the BET surface area, pore volume, and pore diameter of the mesosilica/Pal composite, MCM-41, and Pal. As shown, both the mesosilica/Pal composite and MCM-41 display a type IV isotherm, which is characteristic of ordered mesoporous materials [24]. The pore diameter of MCM-41 was distributed concentrically at 2.6 nm. The pore diameter of the mesosilica/Pal composite was presented at 2.6 and 3.9 nm, respectively, indicating a decrease in the periodic meso-structure of the mesosilica/Pal composite. This result has been confirmed by the small-angle XRD results. The mesopore at 3.9 nm in the mesosilica/Pal composite may be generated by the Pal hard template. The t-plot method was adopted to evaluate the mesopore volume. As such, the exact data of pore volume and pore diameter cannot be calculated for Pal, whose pores are nearly microporous (<2 nm). In conclusion, aside from the micropores in Pal, the mesosilica/Pal composite displayed a micro-mesoporous hierarchical structure. In addition, the BET surface of the mesosilica/Pal composite is calculated at 533.1 cm^2^/g. This is lower than that of MCM-41 (937.2 cm^2^/g). The probable reason for this is the small BET surface of the Pal (only 169.2 cm^2^/g) used in the preparation of the mesosilica/Pal composite samples.

**Figure 3:**
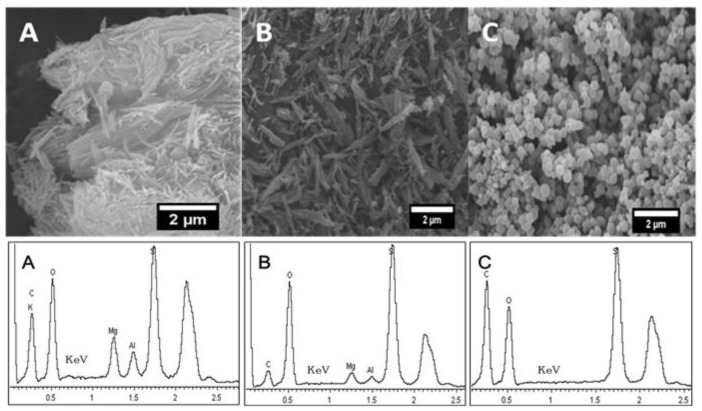
SEM pictures and EDX analysis of (**A**) Pal; (**B**) mesosilica/Pal composite; (**C**) MCM-41.

As presented in Figure 3, **t**he morphologies and elements distribution of Pal, the mesosilica/Pal composite, and MCM-41 were investigated through SEM and EDX. As shown in Figure 3A, Pal crystals are formed by agglomerate nanorods in the range of 2 μm, which is a typical morphology of this mineral. The EDX analysis revealed that there are Mg, Al, K, Si, and O elements in the Pal samples, which is the general composition of a Pal mineral. In Figure 3C, MCM-41 exhibits a typical spherical particle. The particle size is uniform at approximately 200 nm. The element distribution on MCM-41 is very simple, and only includes Si and O. The morphology of the mesosilica/Pal composite is presented in Figure 3B, in which 1D short nanorods that are 200 nm in diameter and 2 μm in length are observed. Compared to Pal original clay, agglomeration for the prepared mesosilica/Pal composite is significantly improved, and all of the nanorod crystals are highly dispersed. In addition, no spherical particle can be observed in the mesosilica/Pal composite sample, indicating that mesosilica is not isolated but generated on the surface of the Pal hard template. The EDX spectra shows that mesosilica/Pal is constituted of Si, O, Mg, K, and Al elements. Except from Si and O which are from the TEOS silicate source and Pal, the remaining metal elements are all from Pal.

**Figure 4:**
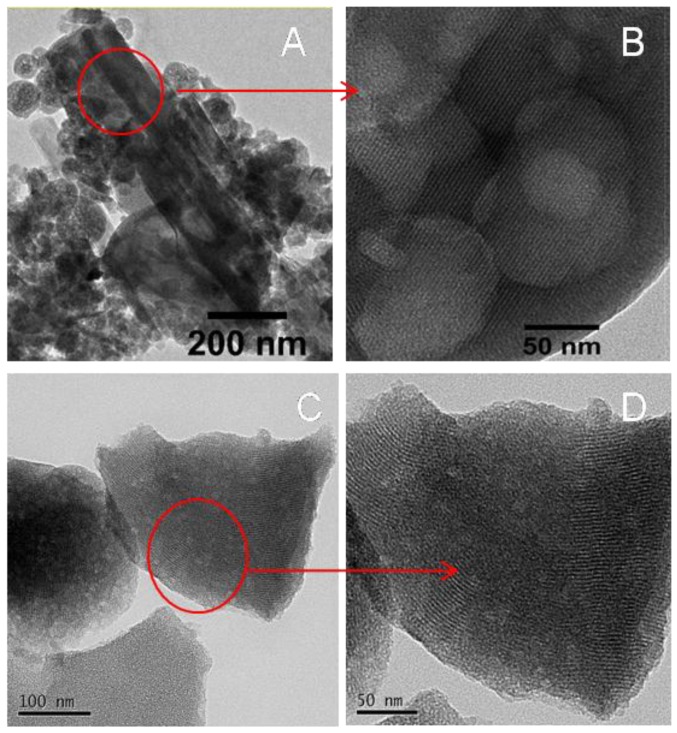
TEM pictures of (**A**) mesosilica/Pal composite-200 nm; (**B**) mesosilica/Pal composite-50 nm; (**C**) MCM-41-100 nm; (**D**) MCM-41-50 nm.

Figure 4A is the TEM image of the mesosilica/Pal composite under the range of 200 nm. As shown, highly dispersed Pal nanorods were wrapped with a layer of silica. To survey the morphology of the silica layer, Figure 4B, which is the amplification of Figure 4A, presents the TEM image of the mesosilica/Pal composite below 50 nm. Uniform long-range mesoporous channels were observed perpendicular to the axial direction. The average diameter of each mesoporous channel was approximately 2–3 nm, further confirming that the mesosilica covered the surface of Pal, and that a composite material was developed. Figure 4C,D presented the TEM images of MCM-41 at 100 and 50 nm, respectively. As shown, the MCM-41 prepared under the same conditions except for the application of the Pal hard template presented a long-term periodic mesoporous structure with a mesopore diameter of 2–3 nm. The obtained results were consistent with the SEM results.

### 3.2. Methyl Orange Adsorption Property

#### 3.2.1. MO Adsorption with Different Materials

**Scheme 2:**
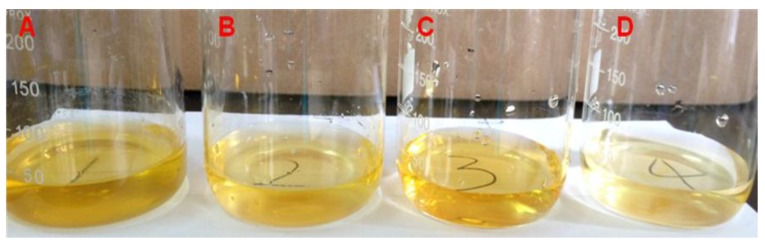
Digital camera images of the materials before and after of the adsorption (**A**): The initial MO solution; (**B**): MO solution after Pal adsorption; (**C**): MO solution after MCM-41 adsorption; (**D**): MO solution after mesosilica/Pal adsorption.

The MO adsorption performances (including q_e_, C_MO_, and A_MO_) of the prepared mesosilica/Pal composite, Pal, and MCM-41 samples are listed in Table 2. Simultaneously, the surface charge of each sample analyzed based on the zeta potential is also exhibited in Table 2. As shown, although MCM-41 has an excellent structure such as a high surface area and long-ranged mesopores for adsorption, its adsorption ratio was as low as 1.8%. It indicated that MCM-41 barely adsorbed MO. It is interesting that Pal, whose effective adsorption surface area was very limited, inversely had a superior adsorption ratio of 35%. Moreover, the good phenomenon is that the adsorption ratio of the mesosilica/Pal composite reached as high as 70.4%, which was much better than that of MCM-41 and Pal. The adsorption performance of these three materials can also confirmed by their digital camera images before (Scheme 2A) and after (Scheme 2B–D) adsorption, as exhibited in Scheme 2. As can be seen, the MO solution becomes nearly transparent after the adsorption of the mesosilica/Pal absorbent. It is suggested that the adsorption performance is not only determined by the pore structure and surface area, but also other factors. Thus, we attempted to analyze the causes of these results from two perspectives. On the one hand, although the mesosilica/Pal composite had less periodic mesoporous channels and a lower surface area than that of MCM-41, the micro-mesoporous hierarchical structure of the composite nanorod enhanced the retention and retarded the migration of MO. On the other hand, the adsorption performance was influenced not only by the structure, but also by the surface charge of the adsorbent. The mesoporous silica had a negative charge density because of the presence of Si–O and Si–OH groups [25]. Therefore, all of these three samples had negative zeta potentials. Among them, the mesosilica/Pal composite had the most positive zeta potential (−4.38 mV). As is known to all, MO (4-dimethylaminoazobenzene-4-sulfonic acid sodium salt) is a typical water-soluble anionic dye that tends to be attracted by positive materials. Therefore, the mesosilica/Pal composite exhibited the superior adsorption performance for MO because of its relatively higher positive surface charge.

We investigated the adsorption performance of some other absorbents such as 700 °C heat-treated Pal [26], acid-treated palygorskite (PAL-O) [20], and Mg–Al layered double hydroxides (LDHs) [27] proposed in the literature. The metal cations played an important bridging effect for the adsorption of anionic dyes MO. Metal cations in absorbents were used as counterions and can be adsorbed to neutralize the free –SO_3_^−^ moieties or other anionic species in the MO. For this reason, the maximum adsorption quantity of MO for Mg–Al LDHs (layered double hydroxides) (molar Mg:Al ratio of 2), much higher than general clay absorbents including the mesosilica/Pal composite reported in this work, was as high as 4.5 mmol/g (about 1471 mg/g).

To study the influency of metal cations in various absorbents, ICP-OES was applied here to investigate the metal atom content and analysis results were exhibited in Table 3. Metals such as Mg, Al, Ca, Fe, K, and Na are rich in Pal. This can be explained as the natural composition of Pal mineral. While in the MCM-41 sample, the metal atom content was lower due to the analytical pure raw material. The prepared mesosilica/Pal consisted of silica coated Pal. As a result, it has more metal atoms than MCM-41 but less than Pal. The ICP analysis results are in accordance with the above EDX results.

#### 3.2.2. Effect of Contact Time on the Uptake of MO by Mesosilica/Pal Composite

**Figure 5:**
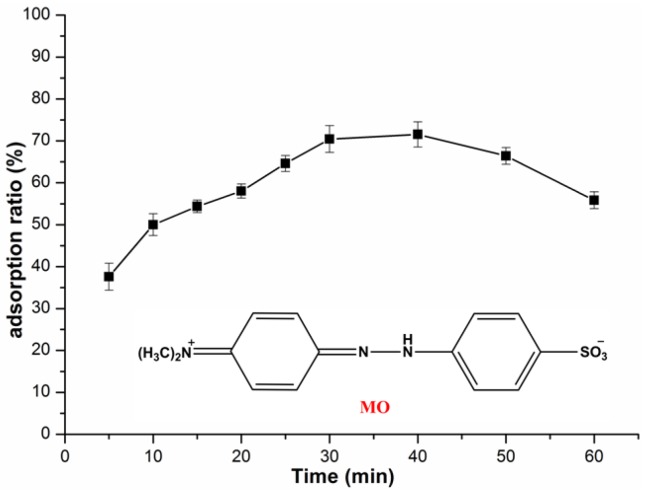
Effect of contact time on the uptake of MO by the mesosilica/Pal composite (under the condition of 298 K, pH 6.0, and an initial concentration of MO of 400 mg/L).

To study the effect of different contact times, the adsorption ratio of the mesosilica/Pal composite was determined at varying time intervals while keeping the levels of adsorbents, pH, and temperature fixed. As presented in Figure 5, the adsorption experiments for evaluating the contact time were conducted with the time ranging from 5 min to 60 min. The adsorption ratio of MO increased rapidly in the initial 30 min and was almost unchanged for 30–40 min, indicating an equilibrium state. A 70% adsorption was reached after only 30 min, and approximately 50% of the MO was adsorbed in 10 min in the case of the mesosilica/Pal composite adsorbent. Such an adsorption performance is significantly superior to that of the other adsorbents, which typically require several hours to achieve equilibrium [28]. The rapid adsorption rate may be attributed mainly to the electrostatic interactions. This result is encouraging because the equilibrium time is an important parameter for waste water treatment. However, after the adsorption equilibrium was attained, the percentage removal of the adsorbate decreased during the next 20 min. The probable reason for this is that the initial concentration of MO significantly affected the MO adsorption. For a given adsorbent dose, the total number of available adsorption sites was fixed. The ratio of the initial mole numbers of MO to the available surface area was decreased. Thus, the percentage removal of the adsorbate decreased as the initial adsorbate concentration was reduced.

#### 3.2.3. Effect of pH on MO Removal by Mesosilica/Pal Composite

**Figure 6:**
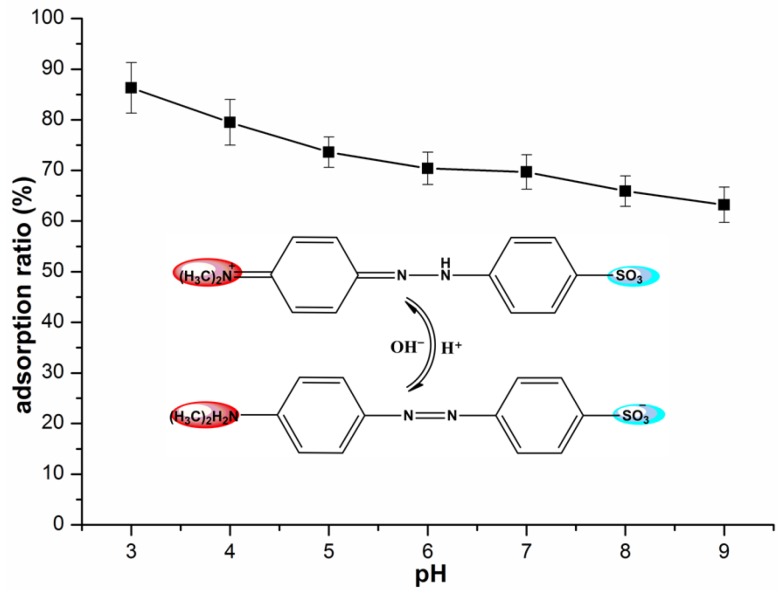
Effect of pH on the uptake of MO by the mesosilica/Pal composite (under the condition of 298 K, 30 min, and an initial concentration of MO of 400 mg/L).

As shown in Figure 6, the effect of pH on the MO removal by the mesosilica/Pal composite was investigated from pH = 3 to pH = 9. As can be seen, a higher adsorption ratio was found at lower pH values. The adsorption ratio is as high as 87% when the pH is 3. It is because of the protonation properties of the adsorbent with a silica skeleton [29]. Lower pH values resulted in higher hydrogen ion concentrations. The negative charges on the surface of the internal pores (as displayed in Table 3, the zeta potential of mesosilica/Pal composite −4.38 mV) were neutralized, and additional adsorption sites were developed because the surface provided a positive charge for the adsorption of the anionic MO [30].

#### 3.2.4. The Regenerability of Mesosilica/Pal Composite

**Scheme 3:**
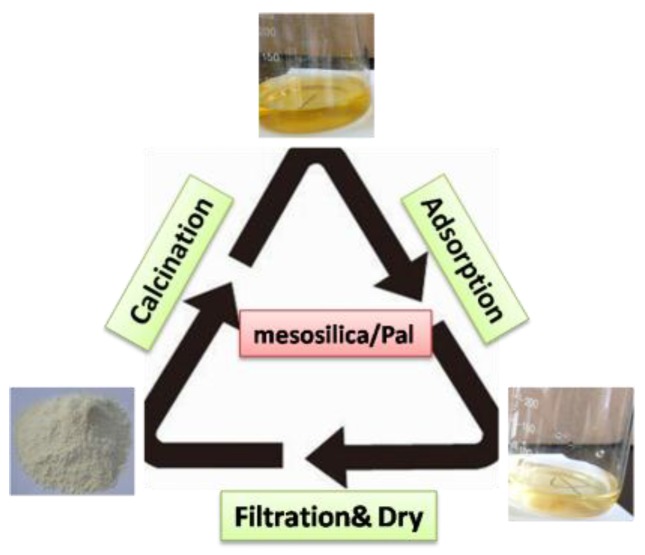
Schematic illustration of the regeneration process of the mesosilica/Pal composite.

**Figure 7:**
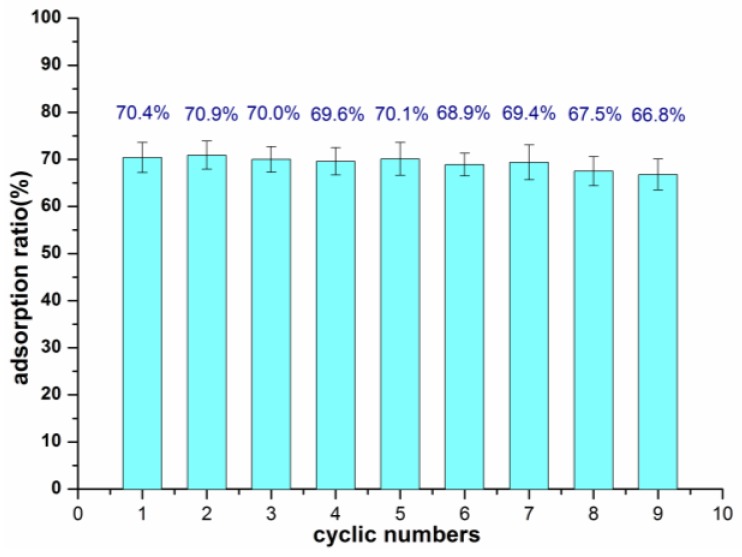
The regenerability of the mesosilica/Pal composite for MO adsorption (under the condition of 298 K, 30 min, pH 6.0, and an initial concentration of MO of 400 mg/L).

To explore the potential regenerability of the mesosilica/Pal composite as an adsorbent for MO removal, thermal treatments were conducted by calcining the exhausted adsorbents at 500 °C for 2 h in air. The specific regeneration process was illustrated in Scheme 3. The MO adsorption performances of the regenerated mesosilica/Pal composites are displayed in Figure 7. As shown, the adsorption ratio of the regenerated mesosilica/Pal composite remains almost unchange for the first five cycles. The thermal regeneration of the mesosilica/Pal composite is further feasible for nine cycles, after which the adsorption capacities of the regenerated materials suffer from progressive reductions of approximately 5% (from 70% to 65%) in comparison with that of the original mesosilica/Pal composite. The superior regeneration performance of the mesosilica/Pal composite is probably due to the high thermostability of the silica skeleton. Progressively decreasing the crystallinity of the mesosilica/Pal composite during the structural reconstruction after thermal treatment may result in decreased adsorption capacities [31].

## 4. Conclusions

A novel 1D hierarchical adsorption material (mesosilica/Pal composite) was prepared through a dual template approach with CTAB and Pal as the soft and hard templates, respectively. The potential of the fabricated composite to remove MO from aqueous solution was assessed. Compared with Pal and MCM-41, the mesosilica/Pal composite was a superior adsorbent for MO removal because of its micro-mesoporous structure and relatively positive surface charge. The adsorption ratio for MO of the composite reached as high as 70.4%, and the adsorption equilibrium time was as short as 30 min. Moreover, the 1D morphology of the mesosilica/Pal composite provided a superior regenerability, and thermal regeneration was feasible for nine cycles.

## Figures and Tables

**Table 1:** The physical chemistry properties of Pal, the mesosilica/Pal composite, and MCM-41.

Material	Pal	MCM-41	Mesosilica/Pal
BET surface area (cm^2^/g)	169.2	937.2	533.1
Pore volume (cm^3^/g)	-	0.77	0.60
Pore diameter (nm)	<2	2.6	2.6, 3.9

**Table 2:** MO adsorption performance and zeta potential of the mesosilica/Pal composite, Pal, and MCM-41 (under the condition of 298 K, pH 6.0, 30 min, and an initial concentration of MO of 400 mg/L).

Materials	Zeta Potential (mV)	* A_MO_	* C_MO_ (mg/L)	* q_e_ (%)	Q_ads_ (mg/g)
Pal	−20.8	0.089	178	35.0	88.80
MCM-41	−24.4	0.135	269	1.8	4.56
mesosilica/Pal	−4.38	0.040	81	70.4	178.61
Heat treated Pal	-	-	-	-	97.80 [25]
Pal-O	-	-	-	-	188.38 [26]
Mg-Al-LDHs	-	-	-	-	1471.00 [27]

* A_MO_: MO absorbance after adsorption determined by a UV-vis spectrophotometer, the error is about ±0.002; C_MO_: MO concentration after adsorption determined by a UV-vis spectrophotometer, the error is about ±2 mg/L; q_e_: adsorption ratios of MO; Q_ads_: adsorption capacity of MO.

**Table 3:** The ICP-OES analysis results of various absorbents.

Materials	Al (ppm)	Ca (ppm)	Fe (ppm)	K (ppm)	Mg (ppm)	Na (ppm)	Ti (ppm)
mesosilica/Pal	15,287.64	3877.91	12,035.59	3038.33	29,620.82	6110.50	5334.48
Pal	23,241.40	8315.25	21,271.64	11,360.13	68,391.34	9435.50	3790.80
MCM-41	9726.40	23.40	−38.51	11.74	16.40	444.50	796.30
